# Vitreous inflammation and macular edema secondary to perfluoro-n-octane toxicity

**DOI:** 10.22336/rjo.2021.35

**Published:** 2021

**Authors:** Fayna Rodríguez-González, Marta Tejera-Santana

**Affiliations:** * Ophthalmology Service, Hospital Universitario de Gran Canaria Doctor Negrín, Las Palmas de Gran Canaria, Spain

**Keywords:** perfluorocarbon, ocular toxicity, intravitreal inflammation

## Abstract

Although it is not frequent, residual perfluoro-n-octane elicits an inflammatory response in form of macroscopic white flake-like material on intraocular structures formed by macrophages with intracellular vacuoles containing it. Macular edema could be another manifestation of this entity which, to our knowledge, has not been described so far. We describe an unusual case of intravitreal inflammation and macular edema secondary to the presence of residual perfluoro-n-octane after a surgical intervention of retinal detachment.

## Introduction

Perfluorocarbon liquids have become widely accepted for use in vitreoretinal procedures especially in complicated retinal detachments surgeries. Their special characteristics such as good visualization, high specific weight, and good surface tension, make them an excellent tool that facilitates surgical procedures. Of them, perfluoro-n-octane liquid has the lowest viscosity, offering low resistance to both injection and aspiration. Despite the stability of perfluorocarbon liquids, several studies have demonstrated toxicity in diverse ocular structures; if they remain retained in the vitreous cavity, they can cause some unusual complications such as in our case. Only a few cases of macroscopic inflammatory reaction, presenting as white flake-like material in intraocular structures, have been reported. We report a case in which not only this type of inflammatory reaction appears, but also a cystic macular edema develops, a situation not described in literature until now.

## Case History

**Fig. 1:**
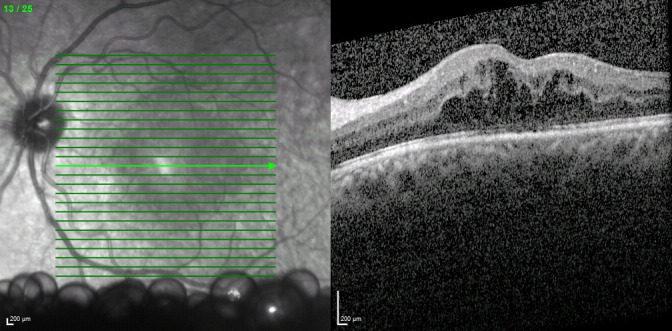
Optical coherence tomography of the left eye. On the left, infrared image showing perfluorocarbon bubbles in the vitreous cavity. On the right, tomographic section showing macular edema

A 54-year-old male developed a rhegmatogenous retinal detachment in his left eye after a cataract surgery. The primary procedure involved 360-degree buckling implant and 23G pars plana vitrectomy following the usual technique. Perfluoro-n-octane liquid was used for reapplication of the retina and was subsequently removed to leave 20% sulfur hexafluoride as a tamponade. A poor visualization due to media opacity was observed in the immediate postoperative period, but apparently all the parameters of the examination indicated normality. However, several days later, the patient presented multiple perfluoro-n-octane bubbles in the anterior chamber and in the vitreous cavity (**Fig. 1**) in addition to a new-onset cystic macular edema. Intraocular pressure at that moment was normal, although in the following days the patient developed intraocular hypertension that was controlled with usual topical and oral medication.

**Fig. 2:**
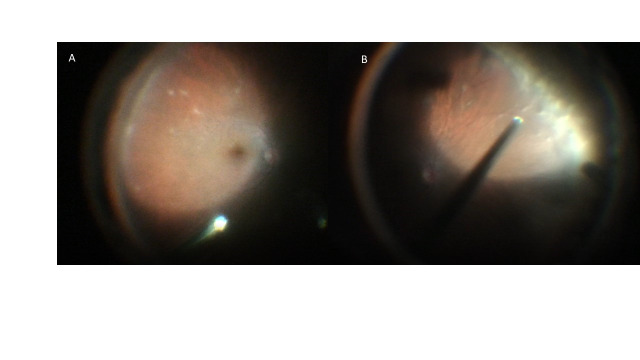
Intraoperative image of the left eye. The inflammatory reaction is observed with the white deposits on the retina. On the left, temporal-lower retina. On the right, nasal periphery

**Fig. 3:**
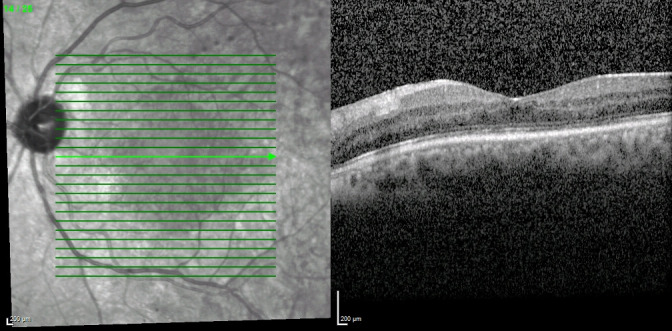
Optical coherence tomography of the left eye forty-eight hours after the intervention showing complete resolution of the macular edema

A new surgical intervention was scheduled for the withdrawal of perfluoro-n-octane, which was carried out several weeks after the initial intervention. During the procedure, it was observed that the retina was applied, but significant vitreous inflammation was identified with abundant macroscopic flake-like material on intraocular structures such as the base of the vitreous, pars plana, and nasal and temporal-lower retina (**Fig. 2**). Due to the inflammatory macular edema, a dexamethasone implant was placed at the end of the surgery. Two days after reoperation, we found an inflammatory reaction with double-cross tyndall effect and some remnants of the inflammatory whitish material on the anterior chamber examination, and an almost complete absence of such inflammation in the fundus, except for few remnants in the temporal-superior periphery. There was complete resolution of the macular edema (**Fig. 3**) and the intraocular pressure improved, but it continued to require topical treatment. 

## Discussion

Perfluorocarbon liquids have become widely accepted for use in the management of retinal detachment surgeries. Their characteristics of low viscosity, good visualization, high specific weight, and good surface tension make them a very valuable tool when performing this type of surgery. Although their full removal is recommended at the end of the procedure, in some cases they have been used as a buffer for limited periods of time [**1**-**3**]. When compared to other perfluorocarbons, perfluoro-n-octane has the best visualization interface and the lowest viscosity, therefore offering less resistance to both injection and aspiration, an important aspect to highlight with the development of surgeries through instruments of ever smaller caliber [**4**]. Despite these characteristics, there are cases of incomplete removal of the perfluorocarbon after fluid-air exchange, especially in cases of poor visualization, just as it happened in our patient. This occurs in approximately 1% to 3.5% of the eyes [**5**,**6**]. The permanence of perfluoro-n-octane for prolonged periods of time has rarely shown, as it occurred in our patient, the development of inflammatory precipitates with a whitish flake-like appearance at the perfluorocarbon-vitreous interface. These precipitates develop mostly in areas where residual vitreous is present [**7**]. If this response is perpetuated, it can lead to more severe cases of subretinal, retrocorneal, or epiretinal membrane formation. Young patients, remaining vitreous, and amounts greater than 0.25 ml of residual perfluorocarbon are possible risk factors for the development of these complications [**8**,**9**]. In our patient, another undescribed complication also appeared, cystic macular edema, which was satisfactorily resolved with surgery and the implantation of an intravitreal dexamethasone device. Given the absence of publications in this regard, we wonder if the perfluorocarbon removal surgery would have been enough to improve this macular edema, although, probably, it would have occurred more slowly and gradually, and not as quickly as in our patient, in which it was resolved after forty-eight hours.

**Conflict of Interest statement**

Authors state no conflict of interest.

**Informed Consent and Human and Animal Rights statement**

Informed consent has been obtained from all individuals included in this study.

**Authorization for the use of human subjects**

Ethical approval: The research related to human use complies with all the relevant national regulations, institutional policies, is in accordance with the tenets of the Helsinki Declaration, and has been approved by the institutional review board of Hospital Universitario de Gran Canaria Doctor Negrín, Las Palmas de Gran Canaria, Spain.

**Acknowledgements**

None.

**Sources of Funding**

None.

**Disclosures**

None.
